# Short-lived mammals (shrew, mouse) have a less robust metal-responsive transcription factor than humans and bats

**DOI:** 10.1007/s10534-016-9926-4

**Published:** 2016-04-11

**Authors:** Katharina Schmidt, Kurt Steiner, Boyan Petrov, Oleg Georgiev, Walter Schaffner

**Affiliations:** Institute of Molecular Life Sciences, University of Zurich, 8057 Zurich, Switzerland; National Museum of Natural History, 1000 Sofia, Bulgaria

**Keywords:** Cadmium toxicity, Longevity, Metal homeostasis, Metal regulatory transcription factor, Zinc-induced transcription

## Abstract

Non-essential “heavy” metals such as cadmium tend to accumulate in an organism and thus are a particular threat for long-lived animals. Here we show that two unrelated, short-lived groups of mammals (rodents and shrews, separated by 100 Mio years of evolution) each have independently acquired mutations in their metal-responsive transcription factor (MTF-1) in a domain relevant for robust transcriptional induction by zinc and cadmium. While key amino acids are mutated in rodents, in shrews an entire exon is skipped. Rodents and especially shrews are unique regarding the alterations of this region. To investigate the biological relevance of these alterations, MTF-1s from the common shrew (*Sorex araneus*), the mouse, humans and a bat (*Myotis blythii*), were tested by cotransfection with a reporter gene into cells lacking MTF-1. Whereas shrews only live for 1.5–2.5 years, bats, although living on a very similar insect diet, have a lifespan of several decades. We find that bat MTF-1 is similarly metal-responsive as its human counterpart, while shrew MTF-1 is less responsive, similar to mouse MTF-1. We propose that in comparison to most other mammals, the short-lived shrews and rodents can afford a “lower-quality” system for heavy metal homeostasis and detoxification.

## Introduction

Shrews live a life on the fast track. Within 1 or 2 years, these small mammals go from birth to sexual maturity, mate, raise offspring, and die. As a consequence of their high metabolic rate—exemplified by 800–1000 heartbeats/min—shrews have to be almost constantly in search of food. With their sharp teeth they shred their prey, mostly insects and worms. Shrews also display a unique feature among mammals, they are venomous and can poison their prey, which helps them to subdue larger animals such as lizards and rodent pups. Daily shrews devour approximately their own body weight in food (as someone commented half-jokingly, a shrew could starve to death in an extended nap). The common shrew, *Sorex araneus*, has a lifespan of only 1.5 years and weighs a mere 9 g (still a giant in comparison to the smallest mammal, the Etruscan shrew *Suncus etruscus* with an average body weight of 1.8 g). The diet of most bats, which represent a distinct group of animals, also consists mainly of insects. But in marked contrast to shrews, bats are generally long-lived, three to four decades have been recorded for free-living bats of the *Myotis* genus (Wilkinson and South 2002; Podlutsky et al. 2005).

**Fig. 1:**
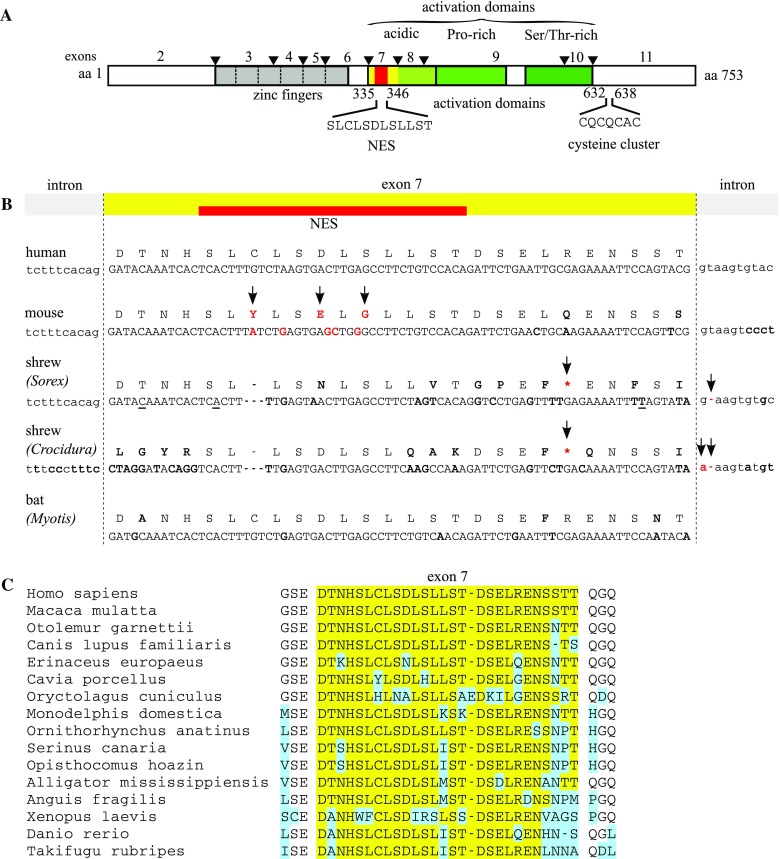
MTF-1 and variants in different species. **a** Schematic view of human MTF-1. Protein-coding exons are numbered 2–11 (the first exon is in the 5′ UTR region, not shown); exon–exon junctions are indicated by *black* *triangles*. The “acidic” activation domain includes exon 7 (in *yellow*) and the nuclear export signal (NES) (in *red*). Other regions critical for metal induction are the zinc finger region, that also harbors the nuclear localization signal, and the cysteine cluster towards the C-terminus (Günther et al. 2012; Suzuki et al. 2015). **b** Exon 7 protein and associated DNA sequences of human, mouse, two distantly related shrew species (*Sorex araneus* and *Crocidura russula*) and bat (*Myotis blythii*). In the mouse, the three amino acids in the NES region that differ from human NES are in *bold red* and further highlighted with *arrows*; replacement of these three by human amino acids converts mouse MTF-1 to high metal responsiveness (Lindert et al. 2009). In both shrews, the 5′ splice site following exon 7 is lost and the entire exon skipped; furthermore, exon 7 is mutated such that the putative reading frame contains a stop codon. These shrew-specific mutations are also highlighted in *red* and with *arrows*; other differences to human exon 7 are indicated in *bold*. In addition there are three polymorphic positions in *Sorex araneus* (*underlined*). Bat intronic sequences were not determined; because exon 7 is present in mRNA, the splice sites must be correct. Exon 7 and NES region are indicated in *yellow* and *red*, respectively. **c** Protein sequence corresponding to exon 7 in other vertebrates. The database was searched for exon 7-like sequences. Representative examples are shown from eutherian mammals (humans, macaque, lemur, dog, hedgehog, guinea pig, rabbit), marsupials (grey short-tailed opossum), monotremes (platypus), birds (canary bird, hoazin), reptiles (alligator, slow worm), amphibians (clawed toad), and fish (zebrafish, fugu). Note that the hedgehog *Echinaceus*, a bigger relative of shrews, contains exon 7. The guinea pig harbors the typical NES alterations of Hystricomorpha-type rodents which prevent robust metal activation, as was shown for capybara (Lindert et al. 2008). Deviations from the human MTF-1 sequence are indicated in *blue*; *gaps* demarcate the boundaries of exon 7 in mammals

We were interested in comparing the activity of the metal-responsive transcription factor (MTF-1) in these two types of mammals—shrews and bats. MTF-1, also referred to as metal regulatory transcription factor or metal response element binding transcription factor, is a key component of the heavy metal regulatory system (Lichtlen and Schaffner 2001; Laity and Andrews 2007; Günther et al. 2012). MTF-1 contains six zinc fingers and several other domains, including ones for nuclear localization, nuclear export and transcriptional activation (Fig. 1a). The DNA binding sites for MTF-1, referred to as metal response elements (MREs) share the core consensus TGCRCNC (R = purine, N = any nucleotide) (Stuart et al. 1985; Wang et al. 2004a). They occur, often in multiple copies, in the enhancer–promoter region of MTF-1 target genes, such as the genes encoding metallothioneins—short, cysteine-rich proteins which avidly bind heavy metals—and some metal transporters (Günther et al. 2012; Laity and Andrews 2007). In the mouse, targeted disruption of MTF-1 leads to death in utero due to liver degeneration (Günes et al. 1998); when MTF-1 is deleted after birth in the liver and partially in the kidney, mice are viable but cadmium-sensitive (Wang et al. 2004b).

The main reason for the present investigation was our previous finding that, in marked contrast to the robust metal responsiveness of human MTF-1, mouse and capybara (a South American rodent) MTF-1s display a poor metal response when tested on MRE-containing reporter genes (Müller et al. 1995; Lindert et al. 2008). Rodent MTF-1s harbor mutations in a region that is important for transcriptional activation by heavy metals and in addition are truncated at their C-termini (Lindert et al. 2008, 2009). This raised the question whether MTF-1 activity reflected the organisms’ needs. Some non-essential, toxic heavy metals can accumulate throughout lifetime, this is especially a challenge for long-lived species—and one major difference between rodents and humans is the much longer lifespan of the latter. Following this vein of thought, we decided to characterize the MTF-1s of the short-lived common shrew *Sorex araneus* and the long-lived *Myotis blythii* (lesser mouse-eared bat). In transfection experiments with a MRE-containing reporter gene, we find that shrew MTF-1 is indeed poorly inducible by zinc and cadmium, due to loss of an entire exon domain involved in transcriptional activation and metal response, while bat MTF-1, similar to its human ortholog, responds well to these heavy metals.

## Results

Exploiting the (incomplete) genome sequence of the common shrew *Sorex araneus* we isolated an MTF-1 cDNA. To our surprise, the shrew transcript differed from all other mammalian MTF-1 mRNAs—lacking a segment encoding 26 amino acids, exactly corresponding to exon 7 (Fig. 1). We considered the possibility that this particular cDNA might represent an organ-specific splice variant, or that the exon had been accidentally skipped in our sample. To address this, we determined the relevant genomic sequence of liver-derived DNA from this shrew and from a representative of another sub-family, the greater white-toothed shrew *Crocidura russula*. White-toothed shrews are separated in evolution by almost 20 Mio years from the red-toothed shrews including *Sorex araneus* (Dubey et al. 2007). The genomic sequence of both species revealed that exon 7 skipping is not accidental, because the splice acceptor site following this exon is mutated and nonfunctional. What is more, the coding sequence is interrupted by a stop codon which would prevent MTF-1 production even if the exon was accidentally spliced-in (Fig. 1b). This finding is remarkable because the skipping of exon 7, while it does not change the reading frame, completely removes the nuclear export signal (NES) and part of the “acidic” activation domain, a region that has been shown to be important for metal responsiveness (Müller et al. 1995; Lindert et al. 2009). In contrast, sequencing of the MTF-1 cDNA from the bat *Myotis blythii* revealed a full-length MTF-1 including exon 7, as is typical for most mammalian MTF-1s. Indeed, an extensive in silico survey of vertebrate genomes including mammals, birds, reptiles, amphibians and fish did not yield any evidence for a deletion of the acidic activation/NES domain (Fig. 1c and not shown). Note that in the NES region, typical mammals are more closely related to birds and reptiles than to rodents (see also (Georgiev et al. 2014)). The MTF-1s of the animals have not been functionally tested, except those of slow worm (Georgiev et al. 2014), and fugu (Auf der Maur et al. 1999), both of which functioned very well in our standard reporter assay (for zebrafish, see also Cheuk (2008)). The extensive alterations in the rabbit *(Oryctolagus)* and the two lysines replacing leucine and threonine in opossum *(Monodelphis)* might be worth a further investigation; both of them are relatively short-lived, rapidly multiplying species.

**Fig. 2:**
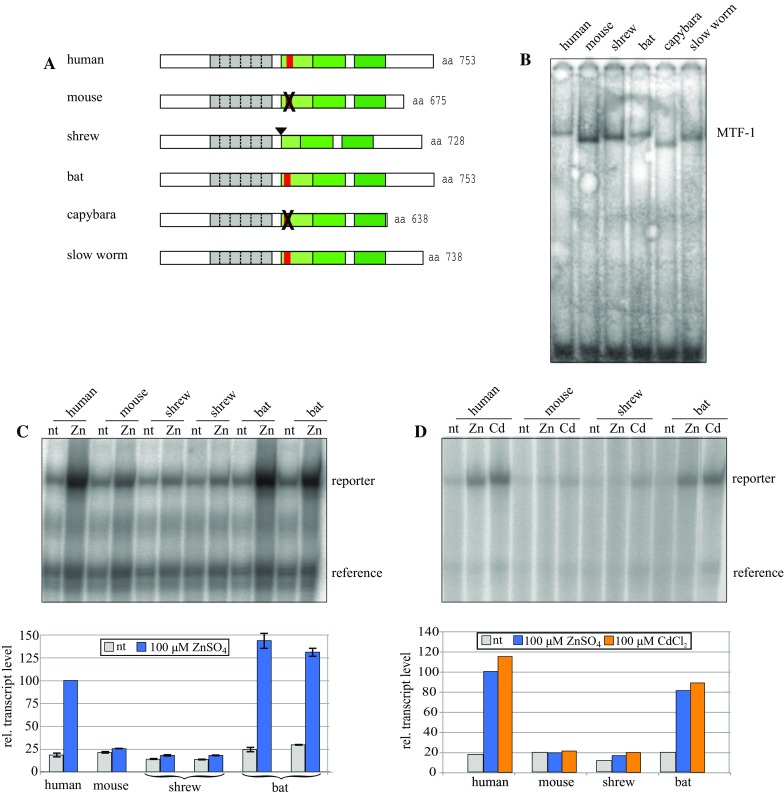
Characterisation of MTF-1 production and activity. **a** Schematic view of MTF-1 proteins used in the experiment: human, mouse, shrew (*S. araneus*), bat (*M. blythii*), capybara (*Hydrochoerus hydrochaeris*, a rodent) and slow worm (*A. fragilis*, a long-lived reptile). The acidic activation domain is indicated in *light green*, the NES motif that is part of it in *red*. The latter is mutated in the rodents mouse and capybara (*black crosses*); in the shrew, the position of the skipped exon 7 is indicated by a *filled triangle*. Note that rodent MTF-1s are also C-terminally truncated. **b** MTF-1 production in transfected cells. Dko7 cells were transfected with expression plasmids encoding MTF-1s of the species shown in **a**. The presence of MTF-1 was verified by EMSA (electrophoretic mobility shift assay) with a labeled MRE consensus oligonucleotide (Westin and Schaffner 1988). **c** Functional comparison of MTF-1s from human, mouse, shrew and bat. Dko7 mouse cells that are null mutant for MTF-1 (Heuchel et al. 1994; Radtke et al. 1995) were transfected with expression plasmids encoding one of the four MTF-1s, together with an MTF-1 dependent reporter gene and a reference gene for normalizing transfection efficiency. Shrew and bat MTF-1 transfections were done in duplicate. Two days later, cells were kept untreated or exposed to zinc sulfate for 4 h and transcripts quantified by the S1 nuclease assay (for details see Methods). The experiment was done twice, the gel of one of them is shown; *error bars* indicate standard deviation of the two experiments. In the bar diagram underneath the corresponding gel, transcript levels with zinc-induced human MTF-1 are set to 100. **d** Experiment similar to **c**, but also including cadmium treatment of transfected cells. The transcript level with zinc-induced human MTF-1 is set to 100. *nt*, no metal treatment

To determine the metal responsiveness of shrew and bat MTF-1s, their cDNAs were cloned into an expression vector driven by the widely used HCMV enhancer–promoter region (Boshart et al. 1985), and tested by transfection into mouse Dko7 cells which lack MTF-1 due to targeted gene disruption (Heuchel et al. 1994; Radtke et al. 1995). Dko7 cells have been used to produce active MTF-1 of various species, from mammals to reptiles to fish (Georgiev et al. 2014; Günther et al. 2012). It should be mentioned that the Dko7 cell clone was established more than 20 years ago; several generations of cells were frozen and used over the years and we note two tendencies: (i) originally transfected mouse MTF-1 induced a 2-3 fold higher transcript level of a 4xMRE reporter when cells were exposed to zinc or cadmium load, while induction by human MTF-1 was 5-12 fold. With the present Dko7 batch, mouse MTF-1 induces only a 1-1.5 fold response upon metal exposure and human MTF-1 3-6 fold. (ii) Early on the basal levels of endogenous metallothionein-1 and -2 transcripts were hardly detectable; now metallothionein-1 RNA is elevated (but still low), perhaps as a compensation for the loss of MTF-1, while the metallothionein-2 gene is completely silenced and cannot be reactivated even with high amounts of transfected MTF-1 (unpublished data). As shown in Fig. 2c, d, human MTF-1 boosted expression of a co-transfected reporter gene approximately 5 fold in response to zinc and cadmium. Bat MTF-1 also responded strongly to these metals, inducing a maximal transcript level similar to its human counterpart. In contrast, both mouse and shrew MTF-1 stimulated reporter gene transcription only marginally in response to metal treatment.

**Fig. 3:**
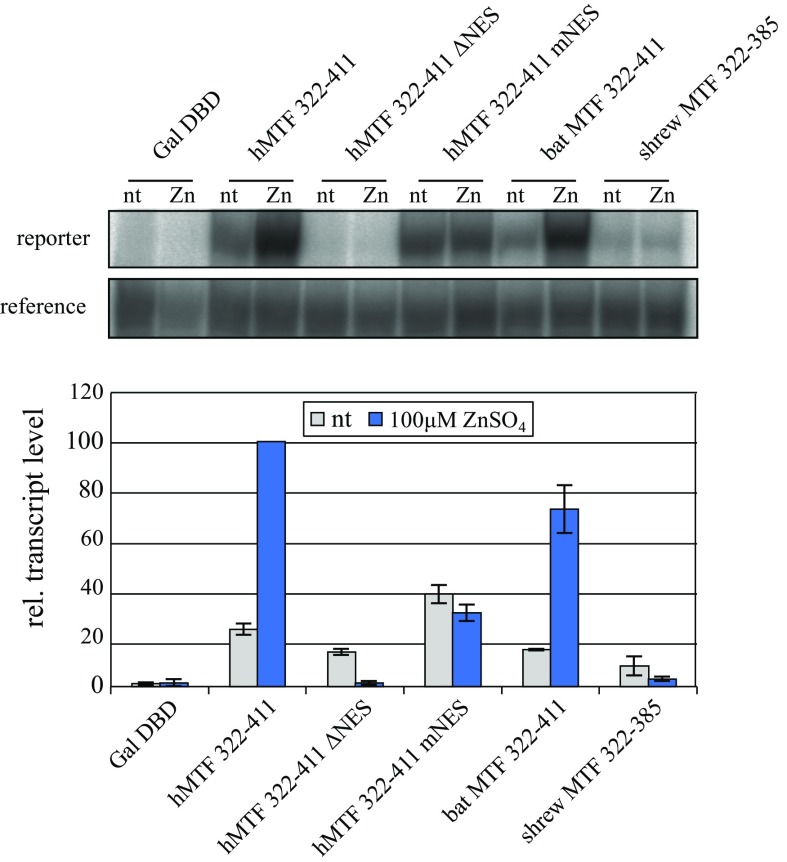
The acidic region harboring the nuclear export signal (NES) is critical for metal response in bat MTF-1. The subsegment encoding the acidic activation/NES region from human, bat and shrew MTF-1s was fused to the DNA binding domain of Gal4 transcription factor. As controls, the human domain was also provided in two mutant forms, one with a deletion of the NES motif and the other with a substitution of its own NES by the one of the mouse, as described in (Lindert et al. 2009). Dko7 cells were transfected with the indicated effector constructs, a reporter containing Gal4 binding sites and a reference gene. Two days later, cells were either kept untreated or treated for 4 h with zinc, and transcript levels were determined by the S1-assay. The *bars* represent the average of two independent experiments; gel bands of one of these experiments are shown above. Transcript levels with the zinc-induced fusion construct Gal4-human acidic domain are set to 100. *nt*, no metal treatment

MTF-1 production in transfected Dko7 cells was verified by EMSA (electrophoretic mobility shift assay, or bandshift) (Fig. 2a, b). We found that all four MTF-1s—human, mouse, shrew and bat—were well expressed. This shows that the low activity of mouse and shrew MTF-1s were not due to a low protein level in transfected cells. Also included were two control MTF-1s, one from the South American rodent capybara (*H. hydrochaeris*) and one from a long-lived European reptile, the slow worm *Anguis fragilis*. With regard to metal induction, the former responds poorly and the latter well (Lindert et al. 2008; Georgiev et al. 2014). Mouse and shrew MTF-1s were particularly well expressed, which in this context is however not important; the overall transcript level of the 4xMRE reporter might be a bit higher but the fold induction was shown to remain constant for a given MTF-1 over an almost 100-fold expression range (Georgiev et al. 2014). Skipping of the entire exon 7 in shrew MTF-1 thus seems to have the same adverse effect as the amino acid substitutions in the same region of the mouse (Fig. 2c, d). To further analyze this phenomenon, we fused a subsegment of MTF-1 spanning the entire acidic activation domain to an unrelated transcription factor, the DNA binding domain (DBD) of Gal4. We had noted before that the acidic activation domain of human, but not of mouse MTF-1, was by itself able to confer metal inducibility to Gal4 (Lindert et al. 2009). The same experimental setting was applied to shrew and bat MTF-1. The fusion constructs were transfected into Dko7 cells and the level of reporter transcripts determined. As shown in Fig. 3, Gal4 fused to the human domain (aa 322–411) yielded a robust response to zinc, which was completely abrogated upon deletion of aa 336–344 in the NES region that is located within exon 7 (see also Fig. 1a). A corresponding segment with a mouse NES, while it displayed an elevated basal activity, failed to induce more reporter transcripts in response to zinc treatment. Not unexpectedly, the activity of the bat acidic activation domain approached the one of its human counterpart, as would be predicted from the long-lived/short-lived hypothesis, while the domain from the shrew, lacking the exon 7 segment, was essentially inactive in this test.

**Fig. 4:**
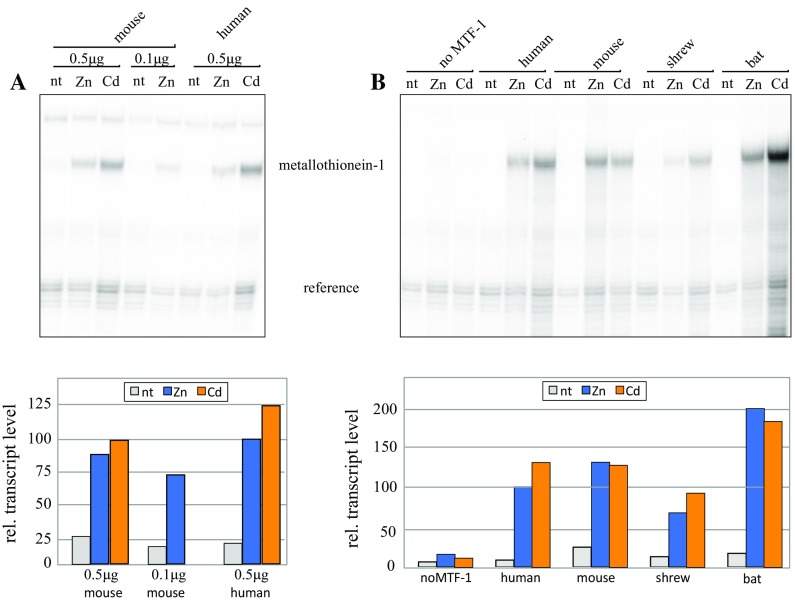
Metal induction of an endogenous metallothionein gene ameliorates interspecies differences of MTF-1 activity. **a** Mouse MTF-1 knockout (Dko7) cells were transfected with mouse and human MTF-1 and transcript levels of the endogenous metallothionein-1 mRNA determined in response to zinc or cadmium load. Transfection efficiency was normalized with a co-transfected reference gene. Besides the standard 0.5 μg of mouse effector plasmid, a lower amount (0.1 μg) was also tested, showing that the concentration of MTF-1 hardly affected metal response (see also Georgiev et al. 2014). The transcript level with the zinc-induced human MTF-1 is set to 100. **b** Experimental setting as in **a** but with MTF-1s of human, mouse, shrew and bat. *no MTF-1*, cells transfected with a reference but no MTF-1 effector gene; *nt*, no metal treatment

Our experiments also addressed an apparent paradox: on the one hand we found weak, if any, metal inducibility of mouse and shrew MTF-1. However, mouse metallothionein-1 and 2 genes contain multiple MREs in their upstream enhancer–promoter region. Moreover, metallothionein transcript levels clearly increase upon heavy metal load (Durnam and Palmiter 1981; Günes et al. 1998). This seems to contradict our earlier observations that not only a 4xMRE reporter but also transfected reporters with genuine metallothionein promoters are poorly and well-induced by mouse and human MTF-1, respectively (Brugnera et al. 1994; Radtke et al. 1995) and unpublished observations). In an attempt to resolve this apparent contradiction, we transfected human and mouse MTF-1 into the MTF-1 null mutant Dko7 cells and determined transcripts of endogenous metallothionein-1, rather than of extrachromosomal reporters. These experiments revealed a striking difference to the previous ones in that metallothionein transcripts were now clearly induced by both, human and mouse MTF-1 (Fig. 4a). The assay was repeated, this time also including dishes without MTF-1, and with MTF-1s of shrew and bat. In cells lacking MTF-1, metallothionein-1 mRNA was present at very low levels and was only slightly elevated by zinc or cadmium load. By contrast, transfection of MTF-1 from each of the four species boosted metallothionein transcripts, whereby shrew MTF-1 displayed the weakest activity (Fig. 4b). Thus the chromosomal context of the metallothionein-1 gene seems to compensate largely, but not completely, for the deficiency of shrew MTF-1 observed in reporter assays.

## Discussion

The fact that mice and shrews are small mammals with a very short life span may well have ramifications for the organismic quality control systems. For example, mouse cells are known to undergo malignant transformation far more readily than human cells; also aging processes are accelerated both at the organismic and the cellular level in the mouse. Are these differences due to specific “programs” or rather the result of a more relaxed quality control in short-lived animals, manifesting itself also in “lower quality” individual proteins? In this context we note that the activity of the mammalian DNA repair protein poly(ADP)ribose polymerase PARP-1 (also referred to as ADP-ribosyltransferase) was shown to correlate with the lifespan of mammalian species. While this effect is mostly due to different expression levels, a higher specific activity of human PARP-1 compared to its rat counterpart seems to also contribute (Grube and Bürkle 1992; Beneke et al. 2010). Here we show a clear difference at the level of a specific regulator protein: MTF-1 of short-lived shrews and mice is less active than MTF-1s from long-lived humans and bats. Regarding the handling of heavy metals, it is conceivable that a less robust system is tolerable for short-lived mammals. For example, the non-essential, highly toxic cadmium has a half-life of 15–20 years in the human body since it is poorly excreted (Jin et al. 1998), which means that chronic accumulation is a particular challenge for long-lived species.

MTF-1 of shrews and mice display defects in heavy metal response that are obvious in standard transfection assays. Of special interest is the fact that the same protein domain has independently been altered in these distinct classes of mammals which are separated in evolution by 100 Mio years. What is more, there are two variants of exon 7 mutations (and C-terminal truncations of the MTF-1 protein) in subgroups of rodents, one shared by mouse, rat and Syrian hamster (suborder Myomorpha), the other by capybara, naked mole rat and guinea pig (suborder Hystricomorpha) (Lindert et al. 2008 and data not shown). We favor the explanation that the acidic/NES domains went defunct “by neglect”, i.e., lack of positive selection, because they were not important for short-lived animals. However, we cannot exclude that the exon 7 region was subject to negative selection in these animals because it constituted an “Achilles heel” for an infectious agent that exploits MTF-1. Viruses, for example, often use cellular transcription factors for their early gene expression. Other hallmarks of the short-lived rodents and shrews are short gestation and suckling periods—perhaps the increased metal-responsiveness of MTF-1 in long-lived organisms has evolved so that they better regulate zinc supply for their offspring during gestation. It would be interesting to test the effect of the various MTF-1s on target genes other than metallothioneins, such as zinc transporters. We also note an exception to the short lifespan stereotype of rodents: the naked mole rat (*Heterocephalus   glaber*) can reach an age of 30 years (Tian et al. 2013) and has a long (70 day) gestation period (Roellig et al. 2011), yet possesses a rodent-specific variant MTF-1. The ancestral rodents may have mutated MTF-1 before this long-lived species evolved and it has found a way to compensate for the defect. It should also be mentioned that rodents are not per se helpless if exposed to heavy metals. As noted above, the transcription of the genes for the metal-binding metallothioneins is stimulated in vivo by metal load.

So why then are the results with transfected reporter genes, whether driven by multi-MRE promoters (or a genuine metallothionein promoter), at variance with the solid response of the endogenous metallothionein gene to both mouse and shrew MTF-1, as shown in Fig. 4? We propose the following scenario: in vivo, MTF-1 in conjunction with a set of cofactors (Günther et al. 2012; Takahashi 2015; Dong et al. 2015; Choi and Bird 2014) ensures metal-induced transcription. The balance of interactions is distorted in transfection experiments with extrachromosomal reporter genes such that only the MTF-1s of humans and bats (and presumably most mammals) are still strongly metal-responsive, thanks to their robust acidic/NES domain. MTF-1s of rodents and shrews that are lacking such a backup system are poorly or not responsive under these conditions. Taken together, we still consider a short lifespan to correlate best with a suboptimally metal-responsive MTF-1.

## Methods

### Cloning of MTF-1 DNAs of bat and shrews

Tissue samples of the shrews *Sorex araneus* (ENST00000373036) and *Crocidura russula* were kind gifts of Peter Vogel (University of Lausanne). From approximately 200 mg tissue, RNA was isolated using Trizol (Invitrogen) following the supplier’s recommendations. MTF-1 cDNA was obtained using the SMART RACE cDNA Amplification Kit (Clontech) and inserted into the same expression vector driven by the human cytomegalovirus enhancer–promoter as described for the MTF-1 clones containing human, mouse, capybara and fugu MTF-1 prepared previously in our lab (Brugnera et al. 1994; Lindert et al. 2008; Auf der Maur et al. 1999; Heuchel et al. 1994; Georgiev et al. 2014). For verification of exon 7 and flanking intron sequences of the two shrews, short genome segments were amplified by appropriate primers. Tissue sampling from *M. blythii* was conducted under License No. 193/1.04.2009 issued by the Ministry of Environment and Water of Bulgaria on the grounds of art. 49, para. 1, item 1 of the Biodiversity Act and art. 3, para. 1, item. 4 and art. 7, para. 1 of Regulation no. 8/12.12.2003. RNA extraction and further processing was done as described above.

### Transfections and transcript quantifications

MTF-1 expression vectors and a reference gene (OVEC-ref) for normalization of transfection efficiency (Westin and Schaffner 1988; Westin et al. 1987) were transfected by the calcium phosphate method into exponentially growing Dko7 cells, which are mouse cells lacking MTF-1 due to a targeted deletion (Heuchel et al. 1994; Radtke et al. 1995). 100 mm dishes were transfected with MTF-1 expression plasmid and OVEC-ref plasmid as described (Brugnera et al. 1994). 16 h after transfection the cells were washed and 43–45 h after transfection incubated for 4 h with or without zinc (ZnSO_4_) or cadmium (CdCl_2_) at the concentrations indicated in the figures, typically 100 μM and 50 µM for zinc and cadmium, respectively. After collection of the cells, RNA was isolated by phenol/DCM extraction; reporter and reference gene transcripts were quantified by the S1-nuclease protection assay as described (Weaver and Weissmann 1979; Westin and Schaffner 1988).

### Preparation of nuclear extracts and electrophoretic mobility shift assay

Exponentially growing Dko7 cells in a 10 cm dish were transfected with 5 µg of MTF-1 of each expression plasmid. Two days later, nuclear extracts were prepared as described in (Schreiber et al. 1989). For the EMSA binding reaction, 6 µg of nuclear extract were incubated with 40 fmol of ^32^P-labeled MRE-s oligonucleotide and samples were separated under non-reducing gel conditions as described (Westin and Schaffner 1988).
